# 

**Published:** 1819-07-01

**Authors:** 


### THE
### Medico-Chirurgical Journal;

OR,

### LONDON

### Medical and Surgical Review:

*( QUARTERLY )*

Exhibiting

A COMPREHENSIVE ANALYTICAL RECORD

OF PROGRESSIVE

MEDICINE & SURGERY;

PECULIARLY ADAPTED TO

THE ARMY, NAVY, COLONIAL, AND COUNTRY PRACTITIONERS.

S.G.O.L
INDEXED

CONDUCTED BY

JAMES JOHNSON, M. D.

Author of "the Influence of Tropical Climates on European Constitutions;"

AND OF

"A Treatise on the Liver, Internal Organs, and Nervous System, &c."

ASSISTED BY SEVERAL EMINENT PRACTITIONERS.

VOL. II.

FROM JULY 1819, TO APRIL 1820.

SURGEON GEN'L'S OFFICE
LIBRARY.
63390

*LONDON.*

Printed for the EDITOR,

By W. THORNE, Red Lion Court, Fleet Street,

[To whom, or to the Editor or Publisher, Books for Review are to be addressed.]

Sold by BURGESS and HILL, Windmill Street, Haymarket; BALDWIN, CRADOCK, and JOY, Pater-Noster Row; HIGHLEY and SON, and T. and G. UNDERWOOD, Fleet Street; J. CALLOW, Princes Street, Soho; Cox and SON, Borough; ANDERSON, West Smithfield; ADAM BLACK, Edinburgh; HODGES and M'ARTHUR, Dublin; Messrs. TREUTTLE and WURTZ, Paris and Strasburgh.

W1
ME15L

### PREFACE

TO

### THE SECOND VOLUME.

In closing the Second Volume, or first Bienniad of our labours, we can do little more than reiterate our grateful thanks for the flattering reception which the Journal has hitherto received. It would ill become us to boast of that success which is more owing to the indulgence of the Public than to the deserts of the work. A great body of the actual Purchasers of this Journal are conscious that they have not transmitted their names for insertion, as Subscribers. We are bound to respect the reasons which induce them to withhold this public testimony of their approbation, and must be thankful for their tacit, but substantial support. To those who have so promptly honoured us with their names in the infancy and uncertainty of our undertaking, and who, of themselves, extend so wide and authentic a chart of the Journal's circulation, we are under a double debt of gratitude, and will always feel a kind of personal attachment to these our earliest Patrons.

Amicus certus *in re incerta* cernitur. ENNIUS.

That the *desire* to improve, on our part, cannot increase, we may be permitted to declare; but that the *means* of enhancing the value of the Journal will daily augment, we may venture to predict. From circumstances, which it would here be unnecessary to detail, the principal Editor has, and for some time will continue to have, opportunities of personally collecting important facts and observations in one of the first Theatres of Medical Science on the Continent of Europe. The advantages resulting in various ways, from this event, will flow through the Journal to the Profession at large. The work will suffer no interruption on this account; but, on the contrary, it will be cultivated with redoubled ardour, during the period in question. No sublunary vicissitude of fortune, while life is spared, shall ever relax the efforts, or change the objects of this Journal.

Dives, inops, Romæ;—seu fors ita jusserit exul;
Quisquis erit vitæ color ——

Under these circumstances, it is confidently anticipated, that the Patrons of this Work will not only continue that patronage, but contribute, by their individual influence, to the extension of its circulation. Our gratitude will be best expressed by our zeal and exertions in their service, and in the cause of Medical Science in general.

Eodem animo beneficium debetur quo datur. P. Syrus.

### TABLE OF CONTENTS.

### VOLS. I. and II.

### VOL. I. No. 1.

ORIGINAL COMMUNICATIONS.

Dr. Burder on Syphilis 1
Dr. Felix on Plethora 25
Dr. Burnett's Case of Gunshot Wound 28
Dr. Moulson on advanced Pregnancy 31
Case of Abdominal Inflammation 35
Cases of Spasmodic Colic 38
Mr. Sheppard's Case of Twins 39
Dr. Wilson Philip on Concussion 43

BIBLIOLOGY.

Dr. Armstrong on Scarlet Fever, &c. 46
Dr. Percival on Fever 63
Dr. Van Rotterdam on Fever 71
Dr. Robertson on Congestive Fever 73
Dr. Williams on Wilson's Tincture 77
Mr. Astley Cooper on Exostosis 79
Mr. Travers on Iritis 87
Dr. Crampton on Periostitis 95

FOREIGN MEDICAL LITERATURE.

Vialle and Broussais on Excitement 103

Quarterly Periscope 113

PORT-FOLIO.

Bayle on Morbid Anatomy 124
Biot on Wounds of the Intestines 129
Observations on Belladonna 131
Case of Ileus 133
On Catarrhal Disorders 133
Intelligence, &c. &c. 136

### VOL. I. No. 2.

FIRST DEPARTMENT.

Pathological Observations on Hæmorrhoids 141
Dr. Robertson's Case of Enteritis 154
Dr. Tytler on the Bengal Sickness 158
Dr. Johnson on Hydrocephalus 162
Dr. Carbutt's Medical Cases 169
M. Chomel's Case of fatal Vomiting 172
Dr. Lyon on Inflammatory Affections 173
Case of painful Affection of the Bladder 181
Mr. Blacklock on Hydrocephalus 183
M. Richerand's Excision of the Ribs 184

REVIEWS.

Maclean on Epidemic Diseases 187
Dr. Percival on Mania and Epilepsy 190
Dr. Armstrong on Typhus, 2d. Ed. 197
Dr. Cheyne on Melena 200
M. Le Beaume on Air-Pump Baths 203
Dr. Scudamore on Gout 204
Sir E. Home on the Prostate Gland 206
Mr. C. Bell's Quarterly Reports 209
Mr. Hunter on the Venereal 212
Mr. Todd on Hernia 212
Mr. Dickenson on Burns and Scalds 218
Messrs. Young and Thomas on Cancer 219
Dr. Clark's Lying-in Report 220
Mr. Newnham on Inversio Uteri 222

FOREIGN DEPARTMENT.

Bayle and Cayol on Gastric Scirrhus 225
Percy on the Dangers of Dissection 233

Quarterly Periscope 237

PORT-FOLIO.

Mr. Hamilton's Case of Laryngotomy 247
Dr. Davis on Infantile Diseases 249
Mr. Williams's Case of Loss of Sight 253
Nux Vomica in Paralysis 254
Case of Gastrotomy 255
Medical Miscellanies 258—272

### VOL. I. No. 3.

FIRST DEPARTMENT.

Mr. Stilon's Case of Visceral Disease 273
Dr. Porter on Hydrocephalus Acutus 277
Dr. Moulson's Cases of Loss of Power over the Voluntary Muscles 295
Dr. Cookworthy's Case of Extra-Uterine Conception 299
Mr. Niell on Marsh and Bulam Fever 303
Dr. Wight's Anatomical Inquiry concerning the Nature of Fever 311
Mr. Guthrie's Remarks on certain Opinions and Doctrines, relative to the Phagedenic Ulcer contained in Mr. Carmichael's Work on the Venereal Disease 323

BIBLIOLOGY.

Dr. Wilson's Practical Observations on the Action of Morbid Sympathies, as included in the Pathology of certain Diseases 341
Dr. Reid on the Nature and Treatment of Tetanus and Hydrophobia 353
Dr. Balfour's Observations, illustrative of the Sedative and Febrifuge Powers of Emetic Tartar 358
Dr. Bateman's Account of the Contagious Fever of this Country, exemplified in the Epidemic now prevailing in London 360
Mr. Mansford's Inquiry into the Influence of Situation on Pulmonary Consumption, and on the Duration of Life 375
Dr. Wilson Philip's Experimental Inquiry into the Laws of Vital Functions 394
Dr. Ayre's Practical Observations on the Nature and Treatment of Marasmus, and of those Disorders allied to it, which may be strictly denominated Bilious 399
Mr. Carmichael's Observations on the Symptoms and specific Distinctions of Venereal Diseases 418

CATALOGUE RAISONNEE.

Armstrong on Fever—Gray's Supplement to the Pharmacopœias—Stanley's Practical Anatomy—Anatomical Examinations—Adams's Life of John Hunter—M'Kenzie's Lectures on Diseases of the Eye—Burrows's Parish Registers.

FOREIGN DEPARTMENT.

Dr. Majendie on Absorption 431

FOURTH DEPARTMENT.

Observations on the reigning Epidemic, or Typhous Fever 436
Report of the Physicians of the Universal Dispensary for Children, Doctors' Commons 448
Dr. Grattan's Report of the Fever Hospital, Dublin 450
High Operation for the Stone 451
M. Broussais's Conversion from the stimulating to the depletory Practice in Typhus and other Fevers 451
Lectures, New Works, Correspondence, &c. 453—456

### VOL. I. No. 4.

ORIGINAL COMMUNICATIONS.

Dr. Dewar's Account of the wounded in the Battle of Algiers 457
Dr. Davies's Cases and Dissections 472
Dr. Carbutt's Medical Cases 476
Mr. Carmichael on Syphilis 482
Mr Johnson on Rabies Contagiosa 494
Dr. Blaud on Compression of the Carotids 498
Mr. Colville on ascites 509

REVIEWS.

Toxicological Epitome 505
Medico-Chirurgical Transactions 516
Dr. Bradley on Stridor Abdominalis 529
Drs. Cheyne, Stoker, Barker, and O'-Brien, on the Fever in Ireland 541
Dr. Hallaran on Insanity 555
Mr. Brodie on Diseases of the Joints 567
Dr. Armstrong on Puerperal Fever 583

CATALOGUE RAISONNEE.

Medical Botany—Hospital Pupil's Guide—Venus sine Concubitu—Mills on the Brain—Porter on Fever—Gregory on Dropsy 601

FOREIGN DEPARTMENT.

M. Rostan on Aneurism of the Heart 603
Semeiological Observations 608

PORT-FOLIO.

Dr. Yeats on Hydrocephalus 611
Dr. M'Whirter's Surgical Operation 614
Cases of Erysipelas Contagiosa 615
Excerpta Epistolaria 617
Correspondence, &c. 620

### VOL. II. No. 5.

BIBLIOLOGY; OR, ANALYTICAL REVIEWS.

Mr. Rennell on Scepticism 1 to 7
Dr. Crampton's Clinical Reports on Dropsies 8—25
Mr. Ballingall on Fever, Dysentery and Liver Complaints 26—46
Medico-Chirurgical Transactions, Vol. IX. 47—52
Mr. Todd's Surgical Report, Richmond Hospital 52—57
Dr. Vose on Hydrocephalus, &c. 57—58
Dr. Duncan's Clinical Reports on Fever 58—64
Dr. Clutterbuck on the Epidemic Fever 64—80
Dr. Percival on Typhous Fever 80—83
Professor Hildenbrand on Typhus 83—86
Sir William Adams on Cataract 86—98

CATALOGUE RAISONNEE.

Dr. Yeats on Water in the Brain 98
Dr. Douglas on spontaneous Evolution of the Fœtus 100
Mr. M'Kenzie on the Lachrymal Organs 101
Mr. Parkinson on Parochial Fever Wards 103
Sir Arthur Clarke on Bathing 104
Dr. Dickson on Houses of Recovery 104
A Letter to Mr. Rennell on Scepticism 104
Mr. Kirby's Surgical Observations 105

FOREIGN DEPARTMENT.

On Ingurgitation, and a convulsive Species of Ebriety 106—114
Dr. Vaidy on Local Blood-letting in various Diseases 114—116

PRACTICAL ESSAYS AND COMMUNICATIONS.

On Phlegmasia Dolens in the Puerperal, Gravid, and unimpregnated States. By Dr. Dickson, of Clifton 116—131
Mr. King's Case of Hystero-Thecotomy 132—133
Dr. Johnson's Account of Dr. Primrose Blair, with the remarkable Appearances on Dissection 133—136
Dr. Carbutt's Cases of Morbid Anatomy 136—137
Mr. Norman on the Danger of suppressing accustomed Evacuations, &c. with Cases 138—141
Dr. Kennedy on Hydrophthalmia, with Cases 142—147
Dr. Mott's Case of Ligature of the Innominata 147—150
Dr Davis's Report of Diseases in the Universal Dispensary for Children, St. Andrew's Hill 151—156
Mr. Dendy's Surgical Report 157
Baron Larrey on the Language of Complaint in Pain 158—162
Medical Intelligence.—Hunterian Society.—Widows of Medical Officers.—Sir William Adams.—Dr. Davis.—Associated Apothecaries.—Correspondence.—New Subscribers, &c. 162—168

### VOL. II. No. 6.

ANALYTICAL REVIEWS.

Dr. Baron on tuberculated Accretions of the Serous Membranes 169
Mr. Dickinson on the Yellow Fever 186
Sir Gilbert Blane on Medical Logick 194
Dr. Parry and Mr. Bell on the Circulation 201
Mr. Mansford on Epilepsy 216
Mr. Power on Midwifery 221
TRANSACTIONS OF SOCIETIES.—Dr. Black on Diseases of the Brain, 231.—Dr O'Brien on ditto, 235.—Dr. Grattan on Inflammation, 236.—Mr. Rumsey on Hæmoptysis, 241.—Dr. Stoker on Constipation, 242.—Dr. Stoker on Apoplexia, 244.—M. Maunoir on the Cautery, 248.—Mr. Travers on Aneurism, 249.—Dr. Archer on Paracentesis Thoracis, 250.—Mr. Carmichael's and Dr. Palmer's Extirpation of the Parotid Gland, 253. Dr. Lecher's Case of successful Cæsarian Operation, 257.—Mr. Chapman's Case of Expulsion of a blighted Fœtus, 259.—Dr. Frizel's Instance of Ruptured Uterus, 260.—Dr Nicholl on Oil of Turpentine Internally, 261.—Drs. Percival and Crampton on Disease of the Pancreas, 263.—Dr. Barry on Intestinal Worms, 266.—Dr. Crampton on Disease of the Mucous Membranes, 267.
Dr. Granville on Prussic Acid 269
Mr. Arnott on Stricture of the Urethra, 273
Dr. Welch on Blood-letting in Fever 278
Dr. Weatherhead on Erysipelas 282
Dr. Majendie on Gravel 287
Mr. Brande on ditto 291
Mr Douglas on the Medical Topography of Canada 293
Dr. Mackenzie on Mineral Waters 296
Mr. Thackrah on the Blood in Health and Disease 296
Mr. Bampfield on Tropical Dysentery 303
Address to the Medical Profession 321
Medical Intelligence, &c. &c. 324-8

### VOL. II. No. 7.

ANALYTICAL REVIEWS.

Dr. Bateman on the Diseases of London, &c. 333 to 357
Dr. Black's Clinical and Pathological Reports 357—367
Dr. Scudamore on Gout, Rheumatism, and Gravel 367—392
Mr. Carpue on the high Operation for Stone 393—400
Dr. Armstrong on Typhous Fever, &c. 401—432
Mr. Wilson's Lectures on the Blood and Vascular System 432—434

TRANSACTIONS OF SOCIETIES, containing, viz.

Mr. Ridley on Berri Berri 435
Dr. Watts on drinking Cold Water 437
Dr. Cheyne, Dr. Foster, and Mr. Williams, on Diseases of the Heart 439
Mr. Todd on Diseases of the Hand and Fingers 444
Mr. Brigham on Pyloric Ulceration 445
Dr. Watts on Suppuration of the Liver 447
Mr. Mantell on Fracture of the Pelvis 448
Dr. Clarke on Leeching in Acute Rheumatism 449
Rusticus on Hydrophobia 450
——— on Hydrophthalmia 451
——— on Hydrops Ovarii 451
Mr. Astley Cooper on Apertures in the Urethra 460
Mr. Kirby on Dislocation of the Shoulder Joint 461
M. Laennec on Diseases of the Chest 494
Mr. Pearkes on Diseases of Sedentary People 495

INTELLIGENCE.

Extract of a Letter from Mr. Jardine 496
Dr. Dickson on Phlegmasia Dolens 497
Mr. Dunglison on Moxa 497
Resolutions of the Surgeon Apothecaries 498
New Publications 501
Correspondence 501
Additional Subscribers 504

### VOL. II. No. 8.

ANALYTICAL REVIEWS.

Dr. Cheyne on Hydrocephalus; M. Vaidy sur L'Hydrocephale; J. F. Coindet sur L'Hydrencephale; Dr. Yeats on Water in the Brain; Dr. Cooke on Hydrocephalus Internus 505
Dr. Jackson on Contagious Fever 529
Dr. Hall on the Mimoses 535
Epidemic Cholera of India 547
Mr. Astley Cooper on Dislocations of of the Ancle-Joint 557
Mr. Wadd's Cases in Surgery, with a Plate 572
Dr. Clark's Medical Notes on Climates 577

SUPPLEMENTAL REVIEW OF PRACTICAL MEDICINE for the first Quarter of 1820 595

Introduction, p. 595.—ART. 1. Mr. Liston on Cephalea, 597.—2. Mr. Wardrop on Ophthalmia, 598.—3. Dr. Bostock on Ophthalmia Periodica, 601.—4. Mr. Simpson on Hemerailopia, 602.—5. Mr. Bell on Diseases of the Teeth, 604.—6. Dr. Hall on Laryngitis, 607.—7. Quadri on Bronchocelé, 610.—8. Kholer on Thoracic Disease, 611.—9. Researches on Ileus, with a Critical Examination of Dr. Abercrombie's Paper on that Subject in the Edinburgh Journal, 613. — 10. Mr. M'Dowal on Peritonitis, 629. — 11. Dr. Barbantini on Lithotomy, 631.—12. Dr. Granville on Anteversio Uteri, 633.—13. Mr. Foot on the Venereal Disease, 634.—14. Mr. Davies on Purpura Hæmorrhagica, 638.—15. Mr. Robinson on Elephantiasis, 641.—16. Dr. Crawford on Eczema Rubrum, 643. 17. Mr. Salter on Chorea, 644.—18. Mr. Allan on Tetanus, 646.—19. Dr. Gower's Portable Sudatorium, 649.—20. Dr. Hunter on Harrogate Waters 650

MISCELLANEOUS INTELLIGENCE.—Tic Douloureux, 652.—Dr. Nuttall's New Arrangement of Diseases, 653.—Palsy and Epilepsy, 654.—Address to the Public 655

APPENDIX, containing Dr. Philip's Correspondence with various Gentlemen on certain Physiological Experiments, 657.—List of additional Subscribers, &c. &c.

### CORRESPONDENCE.

Communications are received from Dr. M'Whirter, Dr. Wight, Dr. Kennedy, Mr. Lanyon, Mr. Brigham, Mr. Niell, Mr. Birnie, Mr. Montgomery, and several others.

☞ A feature of peculiar interest will he added to the next and all succeeding numbers of this Journal.

---

### *Additional Subscribers since last Quarter.*

Addison, Mr. James, Surgeon, Burnham
Allen, Mr. J. Surgeon, Weymouth
Anderson, Mr. Surgeon, Bouverie Street
Andrews, Mr. Surgeon, Richmond
Bath, Jacob, Esq. Surgeon to the Forces
Bean, Mr. Surgeon, Camberwell
Boulton, Thomas, Esq. Thornbury, near Bristol
Clements, Mr. Wadebridge, Cornwall
Coyne Phineas, Esq. No. 9, Edward Street, Portman Square
Crane, William, M. D. Senior Physician to the Boston General Dispensary
Crucifix, Mr. Surgeon, Bouverie Street, Hill Street, London
Dillon, Mr. Surgeon, Lambeth Terrace
Evans, Dr.
Filson, Mr. Robert, Honourable East India Company's Service, Madras
Gilbert, Dr. Theodore, Bermuda
Goolden, R. Esq. Maidenhead
Guthrie, J. G. Esq. Berkley Street, London
Hatfield, Mr. Thomas, Herts
Higgs, Mr. John, Maidenhead, Berks
Hingeston, Mr. John, Surgeon-Apothecary, Coleman Street, London
Hollis, Mr. W. M. Surgeon
Holt, Mr. Surgeon, Tottenham
Illingworth, Mr. William, Surgeon, Hackney
Keith, Dr. James, Trinidad
Kirby, J. Esq. Dublin
Lanyon, Mr. Richard, Lostwithiel
Lithgow, Mr. Andrew, Surgeon, Weymouth
M'Ternan, P. Surgeon, R. N.
Marshal, Mr. Assistant-Surgeon R. N.
Maysmer, Mr. Richard, Surgeon, Ashborne, Derbyshire
Medical Society, Bolt Court
Mitchel, Mr. Surgeon, R. N.
Monteath, Dr. G. C.
Neilson, Dr. Trinidad
Newry Medical Society
Osborne, Mr. Surgeon, Gracechurch Street
Plowman, Dr. Midhurst, Sussex
Quebec Medical Society
Royal Ordnance Medical Society, Woolwich
Russell, Mr. Surgeon, Mount Street
Shaw, Mr. Surgeon, Anatomical Theatre, Great Windmill Street
Shute, Mr. Surgeon, Gosport
Sproule, Dr. Bombay
Stenson, Dr.
Stowe, Mr. William, Surgeon, Buckingham
Tobin, Mr. John, Surgeon
Wight, Mr. Chertsey
Williams, Mr. William, Llandovery, South Wales
Young, Mr. William, Surgeon, Ballymena

